# Cepharanthine Suppresses Herpes Simplex Virus Type 1 Replication Through the Downregulation of the PI3K/Akt and p38 MAPK Signaling Pathways

**DOI:** 10.3389/fmicb.2021.795756

**Published:** 2021-12-09

**Authors:** Yao Liu, Li Chen, Wenjun Liu, Dan Li, Jiuseng Zeng, Qiong Tang, Yuexin Zhang, Fei Luan, Nan Zeng

**Affiliations:** ^1^State Key Laboratory of South Western Chinese Medicine Resources, School of Pharmacy, Chengdu University of Traditional Chinese Medicine, Chengdu, China; ^2^School of Laboratory Medicine, Chengdu Medical College, Chengdu, China; ^3^Department of Pharmacy, Clinical Medical College and the First Affiliated Hospital of Chengdu Medical College, Chengdu, China; ^4^School of Bioscience and Technology, Chengdu Medical College, Chengdu, China

**Keywords:** cepharanthine (CEP), herpes simplex virus type 1 (HSV-1), apoptosis, PI3K/AKT pathways, MAPK pathways

## Abstract

Cepharanthine (CEP) is a naturally occurring isoquinoline alkaloid extracted from *Stephania cepharantha* Hayata. Although its underlying molecular mechanism is not fully understood, this compound is reported as a promising antiviral drug. In the present study, we explore the anti-HSV-1 effects and the underlying molecular mechanisms of CEP *in vitro*. Our results show that CEP could significantly inhibit the formation of plaque and the expression of viral proteins and exhibit a general suppression of replication-associated genes. Whereas HSV-1 infection increases the expressions of phosphoinositide 3-kinase (PI3K), protein kinase B (Akt), and p38 mitogen-activated protein kinase (p38 MAPK) in host cells, CEP was effective indirectly inhibiting phosphorylation levels of the targets in PI3K/Akt and p38 MAPK signaling pathways. Moreover, CEP markedly decreased G_0_/G_1_ phase and increased G_2_/M phase cells and decreased the expression of cyclin-dependent kinase1 (CDK1) and cyclinB1 in a dose-dependent manner. Additionally, CEP increased apoptosis in infected cells, reduced B cell lymphoma-2 (Bcl-2) protein levels, and increased the protein levels of Bcl-associated X protein (Bax), cleaved-caspase3, and nuclear IκB kinaseα (IκBα). Collectively, CEP could arrest the cell cycle in the G_2_/M phase and induce apoptosis in infected cells by inhibiting the PI3K/Akt and p38 MAPK signaling pathways, hence further reducing HSV-1 infection and subsequent reproduction.

## Introduction

Herpes simplex virus type-1 (HSV-1) is a double-stranded DNA virus belonging to the α-herpes virus subfamily that replicates in epithelial cells and produces a lifetime incubation period in neurons. As HSV-1 gets more often reactivated, it has the potential to induce herpetic meningitis and blindness in immunocompromised individuals (Whitley and Roizman, 2001; Wilson and Mohr, 2012). Current treatments for HSV-1 include acyclovir (ACV) and its derivatives, such as famciclovir and valacyclovir, which can inhibit the action of virus DNA polymerase (Vere Hodge and Field, 2013; Kukhanova et al., 2014). However, the number of reports on the resistance of HSV-1 against acyclovir and its derivatives has increased, especially among people with low immunity (Bacon et al., 2003; Morfin and Thouvenot, 2003; Prichard et al., 2011; Watson et al., 2017). Therefore, it is critical to develop new drugs against HSV-1 infection.

Cepharanthine (CEP) is a naturally occurring isoquinoline alkaloid extracted from *Stephania cepharantha* Hayata and demonstrated to have unique anti-inflammatory, antitumor, antivirus, antioxidative, and immunomodulating properties (Rogosnitzky and Danks, 2011; Bailly, 2019). As of the 1950s, CEP has been used to treat various acute and chronic diseases, such as alopecia, leukopenia, xerostomia, and snake bites (Bailly, 2019). Clinically, CEP combinations with antitumor medication are suggested to treat immunosuppression and thrombocytopenia induced by chemotherapy and radiation without obvious side effects. Apart from those mentioned above, there is growing evidence that CEP might be a broad-spectrum antiviral drug that can inhibit infections of SARS, HIV, HSV-1, COVID-19, and Ebola and can treat various autoimmune diseases and allergic reactions due to its immunomodulatory effect (Kim et al., 2019; Rogosnitzky et al., 2020; Li et al., 2021). Therefore, CEP could be considered as a potential therapeutic antiviral drug of interest for treating HSV-1. Our recent study shows that CEP has a significant adverse influence on HSV-1 infection *in vitro*, whereas the underlying mechanism is only preliminarily explored (Liu et al., 2021). To further correlate the antiviral applications of CEP with its underlying molecular mechanisms, the anti-HSV-1 effects, and mechanisms of CEP are explored *in vitro* in this study.

Currently, the epidermal growth factor receptor (EGFR)-phosphatidylinositol 3 kinase (PI3K) signaling pathway may affect multiple steps during herpes simplex virus type-1 infection. At the early stage of the infection, it can change the cytoskeleton and facilitate filopodia formation and membrane fusion, allowing the virus to enter the cells. The PI3K/AKT and p38 MAPK cascade are important signaling pathways in cell proliferation and survival when the virus enters the host cell. They play a vital role in replicating HSV-1 (Johnson et al., 2001; Tiwari and Shukla, 2010). When PI3K/AKT and P38 MAPK are activated, they provide upstream signals to downstream, altering the host cell cycle while eluding the host cell defense system and promoting viral reproduction and spread until the virus completes its life cycle (Ono and Han, 2000; Andrade et al., 2004; Cheng et al., 2020).

In recent years, naturally derived constituents from traditional Chinese medicine have been increasingly gaining popularity for their potential usage as pharmaceuticals or as lead structures to develop novel therapeutic compounds. Considering the multitarget effects of traditional Chinese medicine and the complexity of virus–host interactions, we first used network pharmacology to predict the potential mechanism of CEP inhibiting the HSV-1 infection. The results reveal that CEP influenced cell proliferation and survival by modulating PI3K/AKT and p38 MAPK signaling pathways after infection with HSV-1. However, there is no relevant experimental evidence, and we perform experiments to verify the prediction results.

## Materials and Methods

### Reagents, Cell Lines, Plasmids, and Virus

CEP (19052708, with a purity of 99.08%) was purchased from Chengdu Must Biotechnology (Chengdu, Sichuan, China) and dissolved in DMSO (5 mg/mL) for preservation. Fetal bovine serum (FBS) was obtained from QuaCell Biotechnology (Zhongshan, Guangdong, China). ACV (1411201) was purchased from Qian Jiang Pharmaceutical (Qianjiang, Hubei, China) and dissolved in phosphatic buffer solution (PBS) (10 mg/mL) for preservation. Dulbecco’s modified Eagle’s medium (DMEM) and PBS were obtained from Gibco (New York, United States). Trypsin and penicillin–streptomycin antibiotic were purchased from HyClone (Illinois, United States). Antibodies specific for AKT (C67E7), p-AKTser473 (8200), PI3K (39786), p-PI3K (Tyr458/Tyr199) (E3U1H), Phospho-p38 MAPK (Thr180/Tyr182) (D3F9), cyclin B (sc-166210), Bax (2772), Bcl-2 (D17C4), cdc2 p34 (17):sc-54, and GAPDH (97166) for Western blotting were obtained from Cell Signaling Technology (Danvers, MA, United States). Antibodies specific for gD (sc-21719), gB (sc-56987), and ICP0 (sc-53070) were purchased from Santa Cruz Biotechnology (Santa Cruz, CA, United States). Peroxidase-conjugated goat antirabbit IgG was obtained from ZSGB-BIO (Beijing, China). The cell cycle detection kit (KGA512) and annexin V-FITC/PI apoptosis detection kit (KGA1030) were from KeyGEN (Nanjing, China). HSV-1 was purchased from the Institute of Virology, Medical College of Wuhan University. Vero and Hela cell lines were purchased from ATCC.

### Plaque Reduction Assay

The antiviral activity for CEP was measured by a plaque reduction assay. Briefly, the Vero cells (1 × 10^5^ cells/well) were seeded in a 12-well plate and infected with HSV-1 (MOI = 1.5) at 37°C for 2 h. After removing the inoculum, the infected and non-infected cells were cultured in a DMEM medium containing 2% methylcellulose and 2 × final testing concentrations of the test CEP. After 72 h, the infected cells were fixed with 4% paraformaldehyde (PFA) for 20 min at RT and stained with 1% crystal violet for 30 min.

### Western Blot Analysis

Hela and Vero cells seeded in six-well plates (3 × 10^5^ or 1.5 × 10^5^ cells/well) were exposed to HSV-1 (MOI = 1) for 2 h at 37°C. Then, the inoculum was removed after adsorption, and the cells were treated with serial concentrations of CEP (3, 1.5, 0.75 μg/mL) for 36 h. Cells were lysed in 100 μL RIPA lysis buffer on ice for 30 min and then centrifuged at 12,000 rpm for 15 min. The total protein concentrations in the supernatants were determined using a BCA protein assay kit (P0010, Beyotime, China). After being separated by SDS–PAGE, the proteins were transferred to polyvinylidene difluoride (PVDF) membranes. Then, the membranes were blocked for 1 h with 5% non-fat milk in TBST and incubated with appropriate primary and secondary antibodies for subsequent detection by enhanced chemiluminescence.

### Transmission Electron Microscopy

HSV-1 (MOI = 1) infected Vero cells were treated with CEP (3 μg/mL) for 36 h. The cells were collected when 70–80% of the model group showed obvious cytopathic effects (CPE). Then, they were prefixed with a mixed solution of 3% glutaraldehyde, postfixed in 1% osmium tetroxide, dehydrated in series acetone, infiltrated, and embedded in Epox 812. The semithin sections were stained with methylene blue, and the ultrathin sections were cut with a diamond knife and stained with uranyl acetate and lead citrate. Virus particles in infected cultured cells were observed by transmission electron microscopy (TEM, HITACHI, H-600IV, Japan).

### Real-Time Quantitative PCR

HSV-1 (MOI = 10) infected Hela cells were treated with CEP (3, 1.5, and 0.75 μg/mL) for 24 h. According to the manufacturer’s protocol, total cellular RNA was isolated by TRIZOL (Ambion, United States) reagent, and cDNAs were generated by reverse transcription kit (Tiangen Biotech, Beijing, China). Real-time PCR was performed in triplicate on Bio-Rad using the SYBR green PCR master mix (Tiangen Biotech, Beijing, China). The sequences of primer pairs are listed in Table 1.

**TABLE 1:** Primers for real-time RT-qPCR in this study.

Genes	Forward primer sequence	Reverse primer sequence
HSV-1 ICP0	F: 5′-TGTGCACGGATGA GATCG-3′	R: 5′-TCGTTCACGATCGGG ATG-3′
HSV-1 ICP4	F: 5′-CGACACGGATCCA CGACCC-3′	R: 5′-GATCCCCCTCCCGCG CTTCGTCCG-3′
HSV-1 ICP8	F: 5′-ATGGACAAGGTAA CCATCGG-3′	R: 5′-TTGAAAAACGGAAGG GGGTA-3′
HSV-1 VP16	F: 5′-GGCGTCCTGGATG CTGTGGA-3′	R: 5′-ACTGCATGGAGCCGG TCGTG-3′
HSV-1 ICP22	F: 5′-CGCCGCAGAAGAC CGCAAGT-3′	R: 5′-TGTCGCTGCACGGAT AGGG-3′
HSV-1 UL23	F: 5′-CGATGACTTACTGG CGGGTGT-3′	R: 5′-GCGTCGGTCACGGC ATAA-3′
GAPDH	F: 5′-CAGCCTCAAGATCA TCAGCAA-3′	R: 5′-CCATCACGCCACAGT TTCC-3′

### Cell Cycle Analysis

HSV-1 (MOI = 1) infected Hela cells were treated with CEP (3, 1.5, and, 0.75 μg/mL) for 36 h. The cells were fixed in 70% anhydrous ethanol at 4°C overnight and washed twice with PBS. Then, cells were stained with 500 μL PI/*RNase* staining buffer (KGA512, 20200610) at RT in the dark. After incubation for 40 min, the cell cycle profiles were analyzed by flow cytometry (Cytoflex, Beckman), and the data were analyzed using ModFit LT5.1software.

### Apoptosis Detection Assay

Hela cells in six-well plates were infected with HSV-1 (MOI = 1) and then treated with CEP (3 and 1.5 μg/mL) for 36 h. Cells were collected, followed by trypsinization, centrifuged (250 g for 5 min), and washed twice with PBS. After discarding the supernatant, the cells were suspended with 500 μL binding buffer, incubated with 5 μL Annexin V-FITC and 5 μL propidium iodide (KGA1030, 20201215) for 15 min at RT. Flow cytometric analysis (FCM) was conducted by CytExpert software.

### Network Pharmacology and Molecular Docking

The GeneCards database^1^ revealed an intersection of protein targets using ‘‘Herpes meningitis’’ and ‘‘Herpes Simplex virus type I’’ as the keywords and the targets of CEP. Network visualization of ‘‘ingredient-target-disease’’ was done using Cytoscape (3.7.2). Protein--protein interaction (PPI) analysis was performed using the STRING database,^2^ the organism was limited to ‘‘Homo sapiens,’’ and ‘‘minimum required interaction score’’ was set to 0.04. The Hub gene of CEP for anti-HSV-I was identified by utilizing the ‘‘Network analyzer’’ feature in the Cytoscape program to calculate the degree of PPI. Gene Ontology and KEGG pathway annotation of the Hub gene was performed using the DAVID database^3^ with “Homo sapiens” and “Official gene symbol” utilized. We identify representative enriched top 10 GO terms (BP for biological process, MF for molecular function, and CC for cellular component) and top 10 pathways of KEGG pathway database. All analyses with *P*-values of 0.05 were considered significant, and results were visualized.

TCMSP was utilized to derive the 3-D structure of CEP as a ligand. The receptor for the Hub gene and its PDB ID has been deposited at Uniprot^4^ and the RCSB PDB.^5^ Using the Discovery Studio program, the docking pocket was discovered. We further conducted molecular docking using AutoDock and calculated the consensus score. In general, a three indicates low binding activity, a five indicates moderate binding activity, and a seven indicates strong binding activity.

### Statistical Analysis

All data are representative of at least three independent experiments. All data are expressed as the means ± standard deviations (SD). Statistical significance was analyzed using SPSS 21.0 software using one-way ANOVA with Tukey’s test. *P*-values < 0.05 or < 0.01 were considered statistically significant.

## Results

### Inhibitory Effect of Cepharanthine on Herpes Simplex Virus Type-1 Infection

**FIGURE 1:**
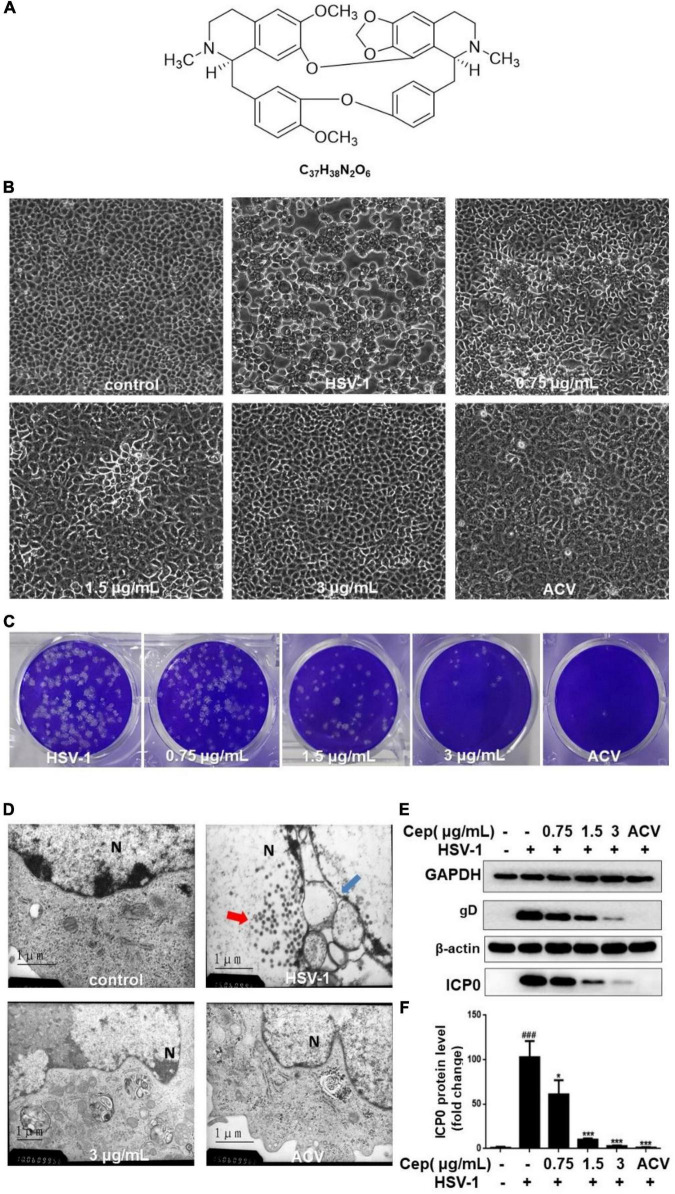
CEP inhibited HSV-1 replication *in vitro*. **(A)** The chemical structure of CEP. **(B)** Vero cells were inoculated with HSV-1 (MOI = 1) and treated with different concentrations of CEP for 36 h, and ACV was used as a positive control. The cytopathic condition of cells was photographed by light microscopy (200×). **(C)** Vero cells were inoculated with HSV-1 (MOI = 1.5) and treated with different concentrations of CEP for 72 h. Plaque experiments were performed to detect the inhibitory effect. **(D)** Vero cells processing HSV-1 (MOI = 1) infection were treated with CEP (3 μg/mL) for 36 h. The transmission electron microscope was adopted to examine the virus particles and cell structure. Red arrows, viruses in the nucleus; blue arrows, disrupted nuclear envelope; N, Nucleus. **(E)** Vero cells in six-well plates were infected with HSV-1 (MOI = 1) in the presence of the CEP at different concentrations, including 0.75, 1.5, and 3 μg/mL for 36 h. Cell lysates were collected for Western blotting as indicated. **(F)** Results from three independent experiments were quantitated and presented as means ± SD. ^###^*P* < 0.001 indicates significant difference vs. NC group; *P < 0.05; ***P < 0.001 indicates significant difference vs. model group.

Our previous experiments confirm that CEP has no obvious cytotoxicity under 3 μg/mL concentration. The median toxic concentration (TC_50_) is 5.4 μg/mL, the median inhibitory concentration (IC_50_) is 0.835 μg/mL, and the therapeutic index (TI) of CEP is 6.47 (Liu et al., 2021). In this study, Vero cells were infected with HSV-1 (MOI = 1) for 2 h and treated with CEP (3, 1.5, and 0.75 μg/mL). The plaque and CPE assays show that CEP could inhibit the plaque-forming and cytopathic effect (Figures 1A–C). TEM results show that CEP promoted the clearance of HSV-1 particles from the cells and maintained the structural integrity of the cell (Figure 1D). Western blot results show that CEP could inhibit the protein expressions of gB, gD, and ICP0 (*P*<0.05) (Figures 1E,F).

### The Inhibitory Effect of Cepharanthine on Herpes Simplex Virus Type-1 Replication-Related Gene Expression

**FIGURE 2:**
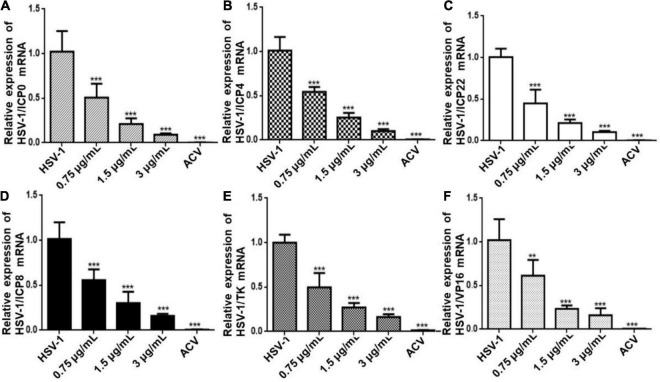
CEP inhibits the transcription of genes associated with HSV-1 replication. Hela cells were mock-treated or treated with CEP at different concentrations, including 0.75, 1.5, and 3 μg/mL for 24 h. The mRNA levels of ICP0 **(A)**, ICP4 **(B)**, ICP22 **(C)**, ICP8 **(D)**, TK **(E)**, and VP16 **(F)** were determined by qPCR analysis and represented as a fold change to mock-treated cells. Results from three independent experiments were quantitated and presented as means ± SD. **P < 0.01; ***P < 0.001 indicates significant difference vs. model group.

To further determine the antiviral effect of CEP on HSV replication, Hela cells were treated with CEP (3, 1.5, and 0.75 μg/mL) for 24 h after infection with HSV-1 (MOI = 10) for 2 h, and the RT-qPCR results show that the expression of immediate early genes (IE) ICP0, ICP4, and ICP22 (Figures 2A–C); early genes (E) ICP8 and TK (Figures 2D,E); and late gene (L) VP16 (Figure 2F) was suppressed after CEP treatment, suggesting that CEP could reduce HSV-1 viral replication in a dose-dependent manner.

### Based on Network Pharmacology to Predict the Potential Mechanism of Cepharanthine Inhibiting the Replication of Herpes Simplex Virus Type-1 Virus

**FIGURE 3:**
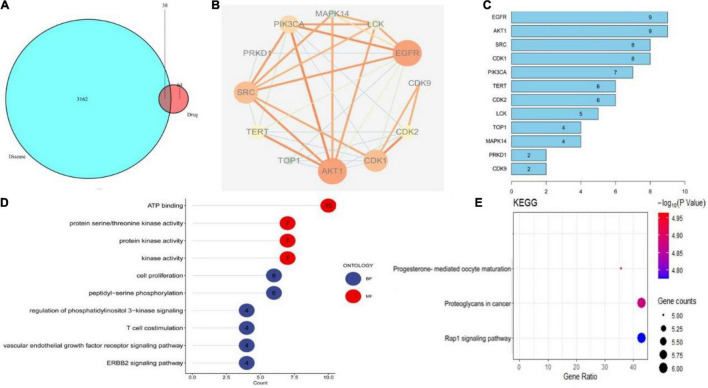
The drug-target interactions predicted by network pharmacology. **(A)** The intersection of drug and disease targets. **(B)** Compound-target-pathway network. **(C)** Protein target interaction (PP1) network of intersecting targets. **(D)** GO terms enrichment analysis (Molecular Function, Biological Process). **(E)** KEGG pathway enrichment analysis.

The primary mechanism of CEP on anti-HSV-1 includes EGFR/PI3K/AKT, the p38 MAPK signaling pathway, and the cell cycle from the findings of the ingredient-target network and PPI analysis (Figures 3A–C). EGFR/PI3K/AKT and p38 MAPK signaling pathways regulate numerous biological processes during HSV-1 infection, including increasing viral entrance and interfering with infected cell proliferation and survival. GO term enrichment analysis reveals that CEP primarily controls cell proliferation, serine/threonine kinase, and energy metabolism (Figure 3D). KEGG pathway annotation shows that the CEP suppression of HSV-1 replication is primarily mediated by the RAP1 signaling pathway, which impacts cell proliferation and survival via regulating PI3K/AKT and p38 MAPK signaling pathways (Figure 3E). As discussed, CEP could influence the proliferation and survival of infected cells through PI3K/AKT and p38 MAPK signaling pathways.

### The Target of Cepharanthine on Inhibition of Herpes Simplex Virus Type-1 Replication

**FIGURE 4:**
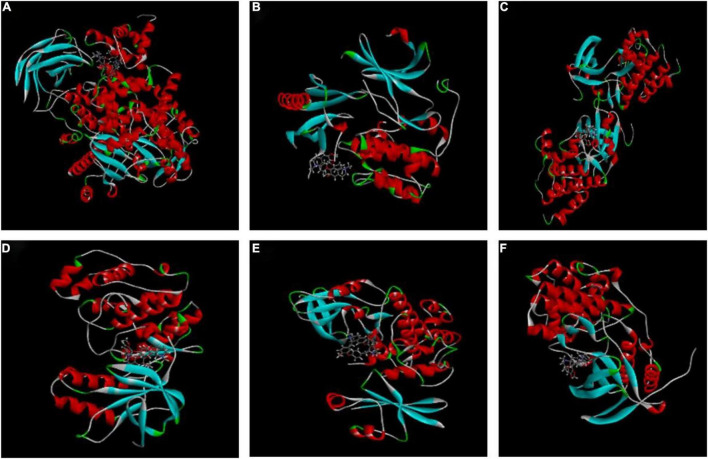
Molecular docking of CEP to potential targets. **(A)** CEP and PIK3CG (3pre). **(B)** CEP and AKT1 (6hhh). **(C)** CEP and SRC (2BDF). **(D)** CEP and P38 (1a9u). **(E)** CEP and CDK1 (6GU6). **(F)** CEP and MAPK14 (5eti).

The higher the LibDockscore, as calculated by molecular docking, the more effectively the ligand binds to the receptor. As shown in Figure 4, MAPK14, p38, SRC, AKT, CDK1, and PI3K all earned a docking score of six, above the threshold value of five, suggesting that CEP induces G_2_/M phase cell cycle arrest and proliferation through modulating the PI3K/AKT and p38 MAPK signaling pathways.

### Cepharanthine Downregulate PI3K/AKT and p38 MAPK Signaling Pathway

**FIGURE 5:**
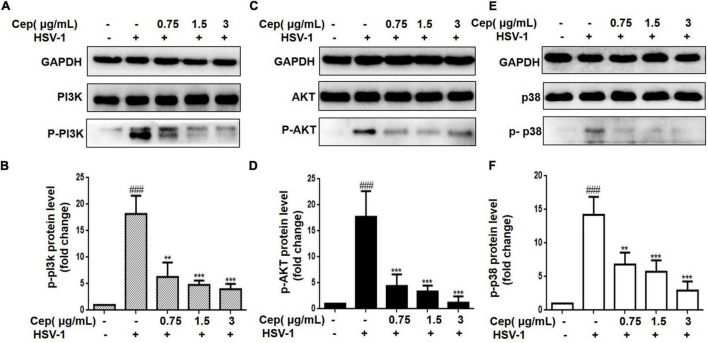
The effect of CEP on PI3K/AKT and MAPK signaling pathway. **(A–C)** HSV-1 (MOI = 1) infected Hela cells were treated with CEP (3, 1.5, and 0.75 μg/mL) for 36 h. Cell lysates were collected for Western blotting as indicated. **(D–F)** Results from three independent experiments were quantitated and presented as means ± SD. ^###^*P* < 0.001 indicates significant difference vs. NC group; **P < 0.01; ***P < 0.001 indicates significant difference vs. model group.

The PI3K/AKT and p38 MAPK signaling pathways are essential for HSV-1 replication and are implicated in numerous aspects of HSV-1 infection. The preliminary results of network pharmacology indicate that the p38 MAPK and PI3K/Akt signaling pathways are the major regulators of cell proliferation and survival after Rap1 activation. To further confirm the anticipated findings, Hela cells were infected with HSV-1 (MOI = 1) for 2 h and then treated with CEP (3, 1.5, and 0.75 μg/mL) for 36 h. We also evaluated the expression of critical proteins involved in the PI3K/AKT signaling pathway. These findings indicate that the PI3K/Akt and p38 MAPK pathways were implicated in the suppression of HSV-1 viral multiplication, whereas CEP could significantly decrease the phosphorylation levels of PI3K in a dose-dependent manner (Figures 5A,D), Akt (Figures 5B,E), and p38 MAPK (Figures 5C,F).

### Cepharanthine Decreases the Cell Population of the G_0_/G_1_ Phase and Increases the S and G_2_/M Phases

**FIGURE 6:**
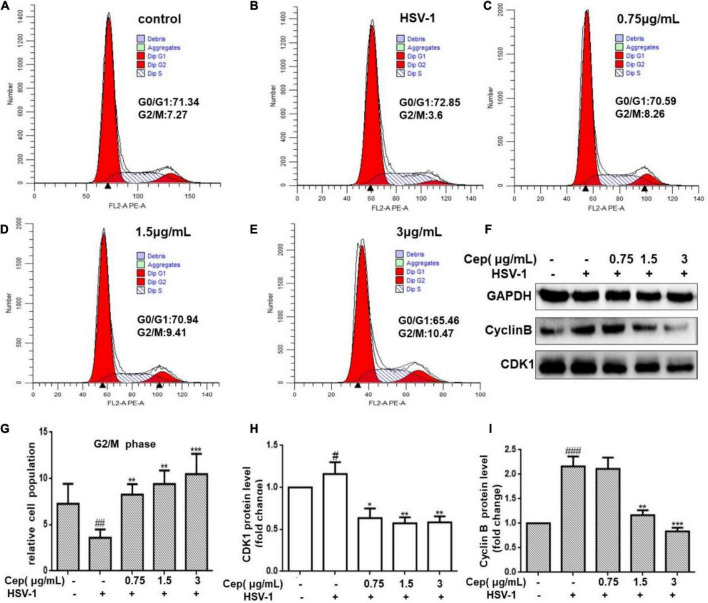
The effect of CEP on the cell cycle of infected cells. **(A–E)** HSV-1 (MOI = 1) infected Hela cells were treated with CEP (3, 1.5, and 0.75 μg/mL) for 36 h. Cells were collected and cell cycle were analyzed by flow cytometry. **(F)** HSV-1 (MOI = 1) infected Hela cells were treated with CEP (3, 1.5, and 0.75 μg/mL) for 36 h. Cell lysates were collected for Western blotting as indicated. **(G–I)** Results from three independent experiments were quantitated and presented as means ± SD. ^#^*P* < 0.05;^##^*P* < 0.01;^###^*p* < 0.001 indicates significant difference vs. NC group; *P < 0.05; **P < 0.01; ***P < 0.001 indicates significant difference vs. model group.

HSV-1 viruses can arrest the cell cycle in the G_0_/G_1_ phase, providing more cellular materials, such as proteins and RNA for their replication, thus establishing an ideal environment for their spread. Hela cells were infected with HSV-1 (MOI = 1) for 2 h and subsequently treated with CEP to validate the cell cycle results predicted by network pharmacology (3, 1.5, and 0.75 μg/mL) for 36 h. FCM and Western blotting were conducted after cell collection. The results show that CEP could markedly inhibit CDK1 and cyclin B (Figures 6F,H,I,F), i.e., decrease the G_0_/G_1_ phase and increase the G_2_/M phases of the cell cycle (Figures 6A–E,G).

### Cepharanthine Promotes Cell Apoptosis of Herpes Simplex Virus Type-1 Infected Cells

**FIGURE 7:**
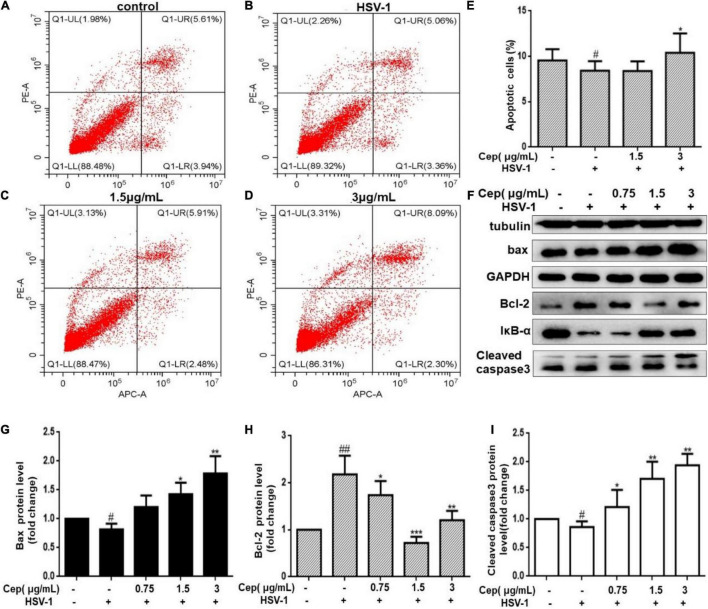
The effect of CEP on the cell apoptosis of infected cells. **(A–D)** HSV-1 (MOI = 1) infected Hela cells were treated with CEP (3 and 1.5 μg/mL) for 36 h. Cells were collected, and cell apoptosis was analyzed by flow cytometry; LL (lower left) represents viable cells (%), UL (upper left) represents necrotic cells (%), LR (lower right) represents early apoptotic cells (%), UR (upper right) represents late apoptotic cells (%). **(E)** Percentages of apoptotic cells in CEP-treated Hela (LR%+UR%). ^#^*P* < 0.05 indicates significant difference vs. NC group; *P < 0.05 indicates significant difference vs. model group. **(F)** Hela cells in six-well plates were infected with HSV-1 (MOI = 1) in the presence of the CEP at different concentrations, including 0.75, 1.5, and 3 μg/mL for 36 h. Cell lysates were collected for Western blotting as indicated. **(G–I)** Results from three independent experiments were quantitated and presented as means ± SD. ^#^*P* < 0.05; ^##^*P* < 0.01 indicates significant difference vs. NC group; *P < 0.05; **P < 0.01; ***P < 0.001 indicates significant difference vs. model group.

Due to the incomplete DNA repair process in the G_0_/G_1_ phase, apoptosis could prevent HSV-1 replication and spread. To clarify that, Hela cells were infected with HSV-1 (MOI = 1) for 2 h and then treated with CEP (3, 1.5, and 0.75 μg/mL) for 36 h. FCM and Western blotting were performed after cells were harvested. The result demonstrates that the number of apoptotic cells was prompted after being exposed to CEP (Figures 7A–E). Western blot analysis reveals that CEP tended to promote the expression of IκBα, Caspase3, BAX, and inhibit the expression of BCL-2 (Figures 7F–I). The survival of cells may be further influenced by apoptosis regulation.

## Discussion

The PI3K/AKT and p38 MAPK signaling pathways are critical in cell survival and can be activated by multiple viruses (Cheng et al., 2020; Zhan et al., 2020), including rotavirus, varicella-zoster virus (VZV), HIV-1, HSV-1, coxsackievirus B3 (CVB3), Epstein–Barr virus (EBV), severe acute respiratory syndrome (SARS) coronavirus, and hepatitis B virus/hepatitis C virus (HBV/HCV) (Rahaus et al., 2004; Lobeck et al., 2016; Niu et al., 2017; Zhang et al., 2018; Torresi et al., 2019; Wang et al., 2019; Bouhaddou et al., 2020). Activated PI3K/AKT and p38 MAPK can mediate apoptosis, cell differentiation, growth, or immune responses (Cooray, 2004; Sun et al., 2015), among which evasion of cell cycle checkpoint regulation and apoptosis inhibition are the major pathways to maintain cell survival and HSV-1 reproduction (Fehr and Yu, 2013; Banerjee et al., 2020). In this study, we first adopted network pharmacology and molecular docking to explore the mechanisms of CEP against HSV-1 replication inhibition. The results indicated that the role of CEP in inhibition of HSV-1 replication is closely associated with the Rap1-related signaling pathway, which is activated by binding with GTP, and its activation mainly affects the cell cycle or apoptosis by regulating the PI3K/AKT and p38 MAPK signaling pathways and thereby affecting cell growth and survival (Raza et al., 2018). In addition, the HSV-1 virus relies on host cells to synthesize large amounts of nucleic acids and proteins to complete the replication and assembly process and produce a progeny virus, during which the GTP is the rate-limiting enzyme for the biosynthesis process and necessary for DNA and RNA synthesis (Jaskiewicz et al., 2018; Shah et al., 2019). Our subsequent experiments demonstrate that CEP inhibits the activation of PI3K/Akt and p38 MAPK caused by HSV-1 infection, but its effect on the cell cycle and apoptosis remain unclear. Considering this, we further investigated the effects of CEP on the cell cycle *in vitro*.

It is reported that the p38 MAPK pathway plays an important role in maintaining cell stay in the G_1_ phase; when host cells are infected with HSV-1, the EGFR and downstream proliferation signals, such as PI3K/Akt and p38 MAPK, encourage resting cells (G_0_ phase) to enter the G_1_ phase (Bulavin et al., 2002; Thornton and Rincon, 2009; Liu et al., 2019). HSV-1 can halt cell cycle progression at the G_0_/G_1_ phase to synthesize large amounts of nucleic acids and proteins required for replication, all while evading the immune response and the G_2_/M phase DNA damage and repair cycle checkpoints, thereby preventing infected cells from dying prematurely (Sanchez and Spector, 2008; Fan et al., 2018). Previous studies demonstrate that PI3K/Akt and p38 MAPK also play a crucial role in the transition between the G_2_ and M phases, and their regulation is largely dependent on the cyclin B-CDK1 complex (also known as the M-CDK complex), which can be targeted directly by viruses to bypass DNA damage-induced G_2_/M phase and DNA repair checkpoints or indirectly via the PI3K/Akt and p38 MAPK signaling pathways (Song et al., 2001; Schang, 2004).

In this study, flow cytometry was utilized to detect cell-cycle changes, and the results find that CEP can markedly decrease the cells in the G_0_/G_1_ phase and increase cells in the G_2_/M phase after HSV-1 infection. To further confirm the findings, Western blot analysis was employed to identify the protein expression of CDK1 and cyclin B, which are important regulators of the G_2_/M phase. These findings show that CEP might activate the host defense system by reducing cell growth by stopping the infected cell cycle in the G_2_/M phase. G_2_/M cycle arrest, on the other hand, may allow for DNA damage repair, which, if unsuccessful, may cause apoptosis and precipitate the death of infected cells to prevent further virus spread in the host. Thus, we sought to elucidate if the processes of apoptosis were involved.

Apoptosis is a form of programmed cell death to maintain cell homeostasis, also a host defense mechanism that limits viral replication and propagation in response to internal stress or bacterial infection. HSV-1 can interfere with apoptotic signaling at multiple levels and prevent infected cells from undergoing apoptosis (Aubert and Blaho, 2001; Nguyen and Blaho, 2006; Guo et al., 2015). An activated PI3K/AKT signaling pathway can inhibit apoptosis by degrading IκBα to activate the NF-κB signaling pathway or promote Bcl-2 protein expression by directly inhibiting caspase-3 (Fei Gross et al., 1999; Chen et al., 2001; Goodkin et al., 2003). Meanwhile, p38 MAPK can affect anti-apoptotic protein Bcl-2 levels through direct or indirect pathways; therefore, Bcl-2 plays a central role in HSV-1 infection-induced apoptosis (Zachos et al., 2001; Huang et al., 2006; Gillis et al., 2009).

Our preliminary results reveal that CEP could induce cell cycle arrest at the G_2_/M phase; however, whether CEP increases apoptosis in infected cells remains unclear as the G_2_/M cycle block can promote DNA repair and apoptosis. In this study, FCM revealed that the apoptosis increased in the high-dose group (3 μg/mL). However, the Western blot results revealed that Bcl-2 protein expression was increased in the group with 3 μg/mL compared with the 1.5 μg/mL group, suggesting that apoptosis is triggered through multiple pathways during HSV-1 infection.

Although this research discusses apoptosis in terms of the PI3K/AKT and p38 MAPK signaling pathways, it is yet unknown if CEP may regulate cell death through other underlying mechanisms during the process of suppressing HSV-1 reproduction. Simultaneously, our previous research shows that CEP may induce autophagy, alleviate endoplasmic reticulum stress, and maintain intracellular homeostasis. Further investigation is needed on the link between these various mechanisms of action and their impact on apoptosis.

## Conclusion

Overall, network pharmacology-based prediction and molecular docking results show that CEP could inhibit HSV-1 replication via the PI3K/Akt and p38MAPK pathways. *In vitro* studies show that CEP suppresses the phosphorylated protein levels of PI3K, Akt, and p38 MAPK. Furthermore, CEP could arrest the cell cycle in the G_2_/M phase and promote late apoptosis in infected cells. In other words, CEP may inhibit the PI3K/Akt and p38 MAPK pathways, which limits HSV-1 viral replication by inhibiting cell growth, promoting apoptosis in infected cells, and inhibiting HSV-1 virus reproduction.

## Data Availability Statement

The original contributions presented in the study are included in the article/supplementary material. The source data for the figures and the data that are not shown are available from the corresponding author upon reasonable request.

## Author Contributions

YL and LC conceived, wrote the manuscript, and designed the study. YL, LC, WL, and YZ contributed to carrying out the experiments. YL, QT, DL, and JZ contributed to data analysis. NZ and FL supervised the research. All authors read and approved the final version of the manuscript.

## Conflict of Interest

The authors declare that the research was conducted in the absence of any commercial or financial relationships that could be construed as a potential conflict of interest.

## Publisher’s Note

All claims expressed in this article are solely those of the authors and do not necessarily represent those of their affiliated organizations, or those of the publisher, the editors and the reviewers. Any product that may be evaluated in this article, or claim that may be made by its manufacturer, is not guaranteed or endorsed by the publisher.

## Footnotes

1
https://www.genecards.org/

2
http://string-db.org/

3
https://david-d.ncifcrf.gov/

4
https://www.uniprot.org/

5
http://www.rcsb.org/
